# Metformin Attenuates Spontaneous and Stimulated Myometrium Contractions in Rat Uterine Strips

**DOI:** 10.5152/eurasianjmed.2025.250630

**Published:** 2025-10-30

**Authors:** Bilge Pehlivanoğlu, Meltem Tuncer, Murat Doğan

**Affiliations:** 1 Department of Physiology, Hacettepe University Faculty of Medicine, Ankara, Türkiye; 2 Department of Surgery, Transplant Research Institute, University of Tennessee Health Science Center, TN, USA

**Keywords:** Contraction, metformin, muscarinic receptors, myometrium, oxytocin

## Abstract

**Background:**

Metformin, an adenosine monophosphate activated protein kinase activator, is indicated in pregnant and non-pregnant women for glucose dysregulation-associated conditions. Its role in various smooth muscle functions was documented. As the myometrium, crucial in fertility and pregnancy, is overlooked, the aim was to investigate the modulation of spontaneous and stimulated myometrium contractions by metformin.

**Methods:**

The uterus of the adult female Sprague–Dawley rats (N = 15) was excised and 4 full thickness myometrium strips (2 × 10 mm) were sectioned. The strips (n = 57) were challenged with KCl (80 mM) to confirm viability and determine the reference maximum response. The spontaneous contractions and dose–response curves for oxytocin (10-11-10-4M) and carbachol (CCh, 10-8-10-4M) were recorded. Additionally, CCh-induced curves were re-obtained in M2-muscarinic receptor blocker, methoctramine (10-5M) and M3-muscarinic receptor blocker, and 4-diphenylacetoxy-N-methylpiperidine (4-DAMP) (10-7M) exposed strips. The responses were obtained alone or in combination with metformin (10-4M).

**Results:**

Metformin attenuated both the amplitude and frequency of spontaneous contractions (*P* < .005) as well as those stimulated by oxytocin and CCh (*P* < .005). This effect was comparable to M2-receptor blockage, whereas the most potent inhibition was with M3-receptor blocker and/or combinations involving it. The 90% inhibition of contraction in metformin, 4-DAMP, and methoctramine exposed strips suggests a synergistic action.

**Conclusion:**

Metformin may be beneficial in conditions favorable to fertilization, implantation, and prevention of preterm labor by reducing myometrial contractility during pregnancy and in non-pregnant indications. However, use of metformin should be approached with caution when strong myometrium contractions are required near term or postpartum.

**Main Points**

- Metformin attenuates the frequency and amplitude of the spontaneous myometrial contractions.
- Metformin attenuates the amplitude of the oxytocin and carbachol-stimulated myometrial contractions.
- Metformin is a good therapeutic candidate for the control of myometrial functions.

## Introduction

Metformin is an oral antidiabetic drug widely used in the treatment of type 2 diabetes mellitus (T2DM) for a long time.^1^ In recent years, its use in obstetrics and gynecological practice has expanded. It has been used in the treatment of polycystic ovarian syndrome (PCOS), for recurrent abortions in PCOS patients, and recently, it has been suggested for the management of gestational diabetes mellitus (GDM).^2^^,^^3^

Although lifestyle modifications including dietary and physical activity measures are sufficient to manage GDM, in a considerable number of patients pharmacological intervention becomes inevitable, since hyperglycemia-associated short- and long-term maternal and fetal adverse effects are well-documented.^2^^-^^5^ The first line of treatment of GDM is insulin, but its various disadvantages including parenteral application, i.e., need for multiple injections, risk of hypoglycemia, maternal weight gain, and higher cost decrease the compliance of the patients to the use of insulin.^3^^,^^5^ On the other hand, metformin offers a good oral alternative that improves hepatic and peripheral insulin sensitivity, providing tight glycemic control for GDM which brings both fetal and maternal benefits.^2^^,^^3^^,^^5^^,^^6^ Even with this beneficial profile, the use of metformin during pregnancy has not gained universal acceptance as it can cross the placenta and could directly alter fetal physiology.^7^^,^^8^ Nevertheless, randomized controlled trials and meta-analysis demonstrated no significant increase in perinatal complications associated with GDM treated with metformin alone or in combination with insulin.^5^^-^^8^ In fact, a considerable amount of data indicates fewer incidences of adverse effects with metformin use during pregnancy.^9^

Metformin is an adenosine monophosphate activated protein kinase (AMPK) activator,^1^^,^^8^ an intracellular signal molecule involved in various pleiotropic effects besides glycemic control. The effect of metformin on various aspects of reproductive function from ovulation to endometrial receptivity has been investigated.^9^^-^^11^ Additionally, its role in modulating smooth muscle functions in various tissues, such as vascular^12^^,^^13^ respiratory,^14^ and urinary^15^ systems has been studied extensively. Interestingly enough, despite the fact that AMPK is involved in the signaling of myometrial contractility, and metformin is indicated in reproductive function associated conditions, its effect on the smooth muscle of the female reproductive system was underexplored.^16^^,^^17^

The myometrium, besides its spontaneous contractions that vary during the menstrual cycle, pregnancy, and post-menopausal period, can be stimulated by parasympathetic innervation and/or agonists.^18^ As the importance of the functions of the thick myometrium layer is critical during gestation, labor, and postpartum periods as well as for the pre-gestational reproductive functions,^17^^,^^18^ the potential effect of any chemical substance, including drugs such as metformin, on myometrium functions is crucial and should be clarified.

In this background, to contribute to a better understanding of its use in reproductive age and obtain clues for possible beneficial effects in GDM and PCOS, the aim was to investigate the modulatory effect of metformin on spontaneous and/or stimulated contractions of myometrium strips.

## Materials and Methods

All animal experiments were conducted in accordance with the National Institutes of Health (NIH) Guide for the Care and Use of Laboratory Animals, and the experimental protocol was approved by the Hacettepe University Institutional Ethics Committee for the Care and Use of Experimental Animals (Approval no: 2011/29-4, Date: 24.05.2011).

Young adult (5-7 weeks old) female Sprague–Dawley rats (n = 15) were housed under standard conditions at 21 ± 2°C and 30%-70% relative humidity with 12-hour darkness/12-hour light illumination (lights were on between 7:00 am and 7:00 pm) with free access to standard rat chow and water, and allowed for a week for adaptation. To minimize the effect of sex steroid hormone variations through the estrous cycle of the animals, the tissue samples were collected at their diestrous phase. The phase of the estrous cycle was followed by both visual examination according to the criteria of Champlin^19^ and microscopic evaluation of the vaginal lavage specimens in accordance with the criteria revised by Cora et al.^20^ The animals were anesthetized with ketamine (90 mg/kg) and xylazine (10 mg/kg) combination. After deep anesthesia was ascertained, the pelvis was opened through a midline incision and both horns of the uterus were excised. In the cold oxygenated Krebs–Henseleit solution (118.4 mM NaCl, 4.7 mM KCl, 1.2 mM KH_2_PO_4_, 1.2 mM MgSO_4_, 25.0 mM NaHCO_3_, 2.5 mM CaCl_2_, 12.2 mM glucose; pH: 7.35-7.40), the uterus horns were cleared of all the surrounding adipose and connective tissues and opened up with a longitudinal incision, 4 full thickness longitudinal strips (10 × 2 mm) were prepared from the anti-mesenteric side of the ovarian pole of the horns, which is more responsive to uterotonic agents.^21^ The strips were mounted vertically in 15 mL double jacketed organ baths containing Krebs-Henseleit solution gassed with a mixture of 95% O_2_ and 5% CO_2 _and maintained at 37°C. Each strip was attached to a force transducer (MAY FDT 05, Commat, Ankara, Türkiye) under a resting tension of 0.5-1 g. Before any experimental procedure, the strips were allowed to equilibrate for at least 60 minutes refreshing the wash buffer every 15 minutes. Spontaneous contractions were recorded for 30 minutes after stabilization. Then, the following contraction paradigms were applied to the strips. One strip in each series was allocated as time-control (n = 15; TC). The mechanical activity was collected real time by a data acquisition/analysis system (BIOPAC MP35, BIOPAC Systems Inc., California, USA).

### Contraction Protocols

In the first protocol, after spontaneous activity was recorded, each strip was challenged for 10 minutes with 80 mM KCl (43.1 mM NaCl, 80 mM KCl, 1.2 mM KH_2_PO_4_, 1.2 mM MgSO_4_, 25.0 mM NaHCO_3_, 2.5 mM CaCl_2_, 12.2 mM glucose; pH: 7.35-7.40) and developed tension was used to assess viability and maximal contraction. After thorough washouts (3 times after the KCl exposure followed by refreshing every 15 minutes), strips were always allowed to rest for at least 60 minutes with washouts every 15 minutes between protocols. Myometrium strips were stimulated by oxytocin (10^−4^ M) or muscarinic agonist carbachol (CCh; 3 × 10^−5^ M) in separate series of experiments in control and metformin (10^−4^ M) pre-exposed strips for 15 minutes following equilibration.

In the second series of contraction experiments (n = 8), a cumulative dose–response curve to increasing concentrations of oxytocin (10^−11^-10^−4^ M) was obtained, then the cumulative dose–response curve to oxytocin was repeated following 15 minutes of incubation with metformin (10^−4^ M). The consecutive doses of oxytocin were applied in 5-minute intervals.

In the third series of contraction experiments, a cumulative dose–response curve with increasing carbachol (CCh) concentrations (10^−8^-10^−4^ M) was recorded where increasing doses were applied in 5-minute intervals. Contraction of the uterine strips to cumulative CCh application was repeated in the presence of muscarinic receptor blockers: selective M_2_ receptor blocker; methoctramine (10^−5^ M), selective M_1_/M_3_/M_5_ receptor blocker; 4-diphenylacetoxy-N-methylpiperidine (4-DAMP; 10^−7^ M), methoctramine and 4-DAMP or metformin (10^−4^ M). Each blocker was applied in conjunction with metformin (10^−4^ M) as the last protocol. The receptor subtypes chosen are determined on the basis of previous reports where the major muscarinic receptors are identified as M_2_ and M_3_ in rat uterus.^22^, ^23^ The initial KCl-induced contraction is considered maximum (100%), and all the other contraction responses are presented as the percent of KCl-induced force.

Each strip was tested with 80 mM KCl after all the protocols were completed, to assure the viability of the strips. Data acquired from strips that are not responding (n = 2) or 15% less responsive to the final challenge with KCl compared to TC strips were excluded (n = 1). The strips were taken out of the tissue baths, sutures were removed, and they were weighed before being discarded appropriately.

### Drugs

All the drugs and chemicals, CCh (CAS No 51-83-2), methoctramine (CAS No 104807-46-7), 4-DAMP (CAS No 1952-15-4), nifedipine (CAS no 21829-25-4), and metformin (CAS NO 1115-70-4) were purchased from Sigma Chemical Company. All of them were diluted in distilled water.

Drugs, solutions, and carriers were freshly prepared and added directly into the bath in order not to exceed the volume of the bath by more than 5%. The concentrations were prepared 100 times more concentrated and applied in 150 μL volume to achieve the desired concentration in 15 mL bath solution.

### Calculations and Statistical Analysis

Data were analyzed by BSL Pro Version 3.6.7 software (BIOPAC Systems Inc., Santa Barbara, CA, USA). The force of contraction was normalized with wet tissue weight (g/100 mg wet tissue weight). Statistical analysis was performed with SPSS 22.0 analysis software (SPSS, Chicago, USA). All data are presented as mean ± SEM. *P *< .05 was considered statistically significant.

Normality testing of the data by the Shapiro–Wilk test indicated normal distribution. Between groups comparisons for spontaneous, KCl- or agonist-induced contractions were carried out by one-way analysis of variance (ANOVA) with post hoc Bonferroni correction. The effects of receptor blockers and/or metformin were evaluated by repeated measures of ANOVA followed by Tukey’s post hoc test, as they were carried out using the same tissue samples.

The maximum response (Emax; maximum force of contraction in response to CCh stimulation) and pEC_50_ (negative logarithm of molar concentration that results in 50% of the maximum response) values were calculated by using Sigma Plot 9.01 for Windows software and evaluated statistically by Student’s *t*-test for independent samples. N and n designate the number of the animals and strips, respectively.

## Results

The average weight of the animals, excised uteri (n = 15), and the strips (Total n = 57, excluding 3 non-responding strips) was 230.0 ± 44.5 g, 357.4 ± 40.2 mg, and 54.8 ± 12.5 mg, respectively.

The amplitude and frequency of spontaneous contractions of the metformin pre-incubated myometrium strips were significantly attenuated compared to control (*P* < .05) (Table 1).

The maximum contraction induced by oxytocin (10^−4^ M) (94.32 ± 5.21 % KCl contraction) significantly attenuated when strips were pre-incubated with metformin (10^−4^ M) (41.32 ± 3.81 % KCl contraction) (*P *< .005). Similarly, the maximum contraction response recorded in CCh (3 × 10^−5^ M) exposed strips was significantly lowered in the metformin group (82.04 ± 3.54 and 64.83 ± 2.75 respectively) (*P *< .05) (Table 1).

**Figure 1.**
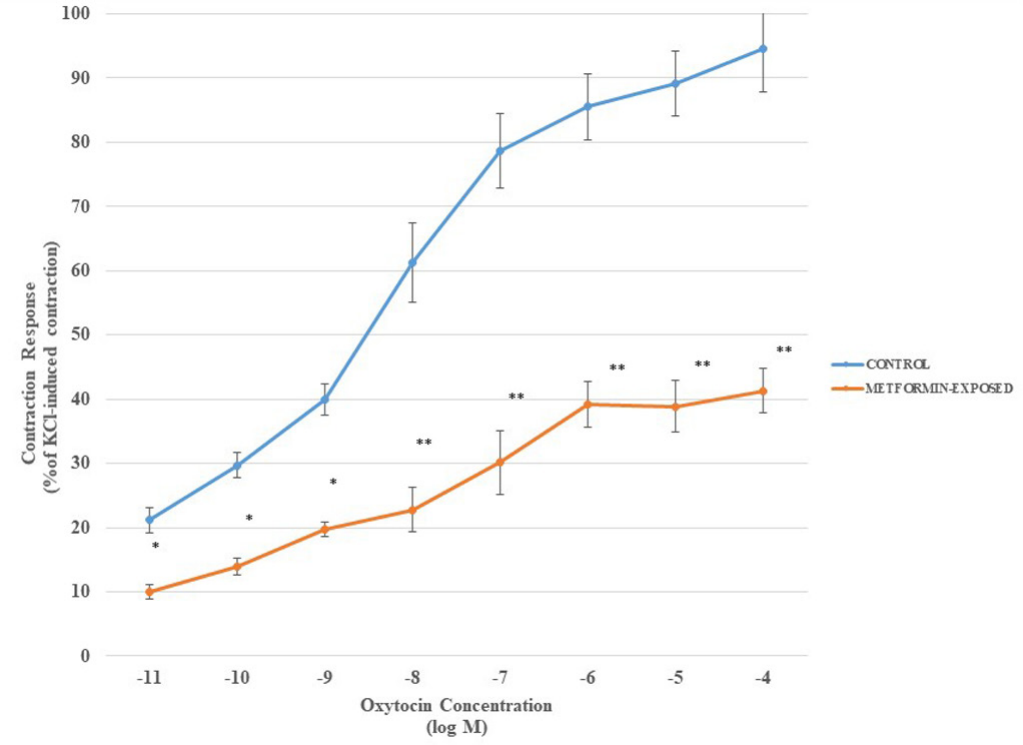
Cumulative oxytocin (10^−11^-10^−4^ M) dose–response curves of myometrium in metformin-exposed and control strips. *P < .05 compared to control, **P < .001 compared to control.

The results of oxytocin dose–response curves in metformin pre-incubated and control myometrium strips are given in Figure 1. The curve shifted to the right in metformin-exposed strips, and this attenuated contraction response to oxytocin became more prominent at the higher doses. The Emax and pEC_50_ values for the agonist are calculated as 94.32 ± 5.21 and 9.53 ± 0.51 and 41.32 ± 3.81 and 4.28 ± 0.31 in control and metformin-exposed strips, respectively (*P*_Emax_ < .001 and *P_pEC50_*< .05).

**Figure 2.**
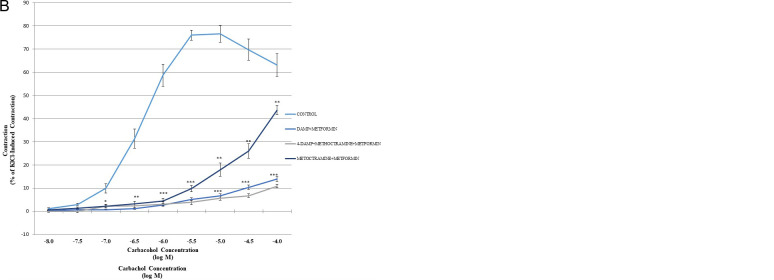
Cumulative dose–response curves of the myometrium to estrous stimulation in: a) metformin, M_2_ receptor blocker, methoctramine, M_3_ receptor blocker, 4-DAMP, methoctramine+DAMP pre-incubated or control; b) methoctramine, 4-DAMP and methoctramine + DAMP together with metformin pre-incubated or control myometrium strips. *P < .05, **P < .001, ***P < .0001 dose points of all groups vs. control, ^§^*P* < .01 dose points of all groups vs. control ^#^*P* < .05 significant difference in carbachol dose–response curve within the same group.

A similar pattern was observed in CCh dose–response curves obtained from control and metformin-exposed tissues (Figure 2A). To investigate the possible involvement of muscarinic receptors in metformin-induced modulation of myometrium contractions, methoctramine and 4-DAMP, the blockers of predominating receptors in the myometrium, M_2_ and M_3_ respectively were used. The cumulative dose–response curves to CCh (10^−8^-10^−4^ M) and its change with muscarinic receptor blockers only or when simultaneously administered with metformin are given in Figure 2A and B. The cumulative application of muscarinic agonist CCh significantly increased the force of contraction in all groups except methoctramine and 4-DAMP combination exposed groups, regardless of metformin application.

Metformin alone shifted the whole CCh dose–response curve to the right and attenuated the maximum contraction response. Similar responses were obtained in M_2_ receptor blocker methoctramine-treated myometrium strips. The difference between metformin- and methoctramine-treated strips was significant at lower doses and became comparable to the control strips at the 2 highest doses of CCh. The contraction response to cumulative CCh stimulation in M_3_ receptor blocker, 4-DAMP, exposed strips was significantly decreased compared to control and metformin and methoctramine-treated strips (*P *< .005). This difference became evident even at lower doses and persisted in all applied doses (Figure 2A).

The use of muscarinic receptor blockers and metformin in combination decreased the force of contraction more prominently. When methoctramine and 4-DAMP or methoctramine, 4-DAMP, and metformin were applied together, almost 90% inhibition in myometrium contraction was achieved. The contraction response when metformin and methoctramine were applied in combination was significantly different from only metformin- or methoctramine exposed strips (Figure 2B).

Emax and pEC_50_ values of CCh in myometrium strips in control and metformin and/or muscarinic receptor blockers pre-incubated groups are given in Table 2.

## Discussion

The results of the present study investigating the effect of metformin on spontaneous and stimulated myometrial contractions provide novel evidence that metformin attenuates the stimulated contractions and provides further evidence regarding its effect on spontaneous contractions of rat myometrium in vitro. These results also indicated that simultaneous exposure of myometrium to predominating muscarinic receptor blockers and metformin silences tissue by more than 90%, suggesting a potential synergistic mechanism.

Although the safety concerns resulted in the withdrawal of metformin in many, although not all, countries,^3^^,^^24^ it has gained increasing acceptance as a safe, effective, and rational option for reducing insulin resistance during pregnancy in patients with T2DM, GDM, or PCOS, it is also reported to provide benefit in non-diabetic obese pregnant women,^1^^,^^2^^,^^7^^,^^24^^-^^30^ recently. As the known and proven effects of metformin are attributed to the activation of AMPK at the molecular level,^1^^,^^8^ it is natural to expect all the physiological processes involving AMPK to be modulated by metformin. Considering the reports indicating a favorable and safe profile of metformin in women, one of its least studied pleiotropic effects, the uterine smooth muscle relaxing effect, was investigated in this study.

Myometrial contractility is crucial not only during gestation and labor but also for the transport of gametes and secretions and embryo implantation, i.e., in the non-pregnant conditions.^27^^,^^28^ Inadequate contraction and/or relaxation may end up in serious fetomaternal outcomes and reproductive system problems.^27^^-^^29^

The spontaneous activity of the myometrium is important, especially for sloughing of the endometrium in the non-pregnant uterus and for the ovum, sperm, embryo transfer, and implantation during fertilization and early pregnancy.^27^^-^^29^ However, after implantation, a silent uterus is critical until term. Among the major complications of clinical conditions where metformin is indicated, such as diabetes or PCOS, failure to implant, abortions, and premature birth can be named.^10^^,^^11^ As noticed, all of them are associated with increased and/or untimely contractions. These results showed decreased amplitude and frequency of spontaneous contractions in metformin-exposed strips. This is one of the very few reports regarding the effect of metformin on myometrium contractions; further investigation in in vivo conditions will add to the advantages of metformin use during pregnancy.

The efficient contractions of myometrium are critical for a healthy delivery, but it is important to have them on time. The regulation of myometrium activity is a complex process of interactions between neurohypophysial hormones, sex steroids, and neurotransmitters; furthermore, involvement of various signal transduction pathways results in timely and precise contraction or relaxation of the myometrium.^25^ Among the uterotonic agents, oxytocin and muscarinic receptor agonists are widely used to investigate the contractility of the myometrium. Accordingly, the effect of metformin on myometrium response to agonist (oxytocin) and neural (CCh) stimulation was studied. The results obtained in this study pointed out the attenuated force of contraction in metformin-exposed strips to both types of stimulation.

The cumulative oxytocin dose–response curve was significantly attenuated in metformin pre-treated strips in all doses. Since the expression of oxytocin receptors was reported to be modulated by steroid hormones changing during the estrous cycle and pregnancy,^30^^,^^31^ to provide standardization of the myometrium responsiveness, samples were taken at the same hormonal stage of their cycle. The only study investigating the effect of metformin on oxytocin-induced contractions revealed no change in myometrium response at term.^16^ Although the results are contradicting, the study by Hehir et al^16^ used cumulative doses of metformin and a single dose of oxytocin which is not a reflection of physiological processes, and myometrium response changes to cumulative stimulation by the agonists. On the other hand, a study investigating metformin and oxytocin interaction in gastrointestinal smooth muscles revealed similar results in counteracting the effect of oxytocin.^32^ This may be explained by the intersection of the metabolic effects of oxytocin and metformin. Oxytocin increases glucose entry to the cells, which in turn changes the status of the cell from energy-deprived to energy-rich and moreover stimulates AMPK, the intracellular signal that carries the action of metformin, too. This fact causes mutual mitigation of the effects of oxytocin and metformin.

The decreased responsiveness to oxytocin suggests that metformin use may facilitate fertilization and contribute to preventing implantation problems, as well as abortion or premature birth-associated morbidity and mortality. However, if the drug of choice is metformin for the medical indications mentioned above, it should be discontinued as the time of delivery approaches. Otherwise, prolonged labor and associated risks become inevitable. The pharmacokinetic studies indicate the elimination time of the active substance as 4-5 days,^33^ that is why it will be a good decision to stop metformin and continue with insulin in pregnant women as soon as the baby is ready to be delivered.

The results about the modulation of muscarinic stimulation of the myometrium by metformin and the contribution of muscarinic receptor blockers indicated a decreased response to cumulative CCh application, similar to oxytocin. However, the effect of metformin was significant at moderate doses and comparable to the control strips at the lowest and highest doses applied. The dose–response curve was shifted to the right as indicated by lower pEC_50_ values. The loss of relaxing effect at higher doses suggests an effective dose window for metformin in regard to the myometrium and should be kept in mind in further studies. The application of M_2_ and M_3_ muscarinic receptor blockers also attenuated the contraction response of the myometrium strips. The most significant effect was obtained in M_3_ receptor blocker exposed strips and accordingly in the tissue samples exposed to the combination of M_2_ and M_3_ receptor blockers, in support of previous data stating M_2_ and M_3_ predominance in uterine smooth muscle cells.^22^^,^^34^

Our results confirmed the well-documented smooth muscle relaxing effect of metformin is valid for spontaneous and stimulated contractions of the myometrium as well. However, the effect of metformin on neural stimulation is comparable to methoctramine but not as potent as DAMP.

Further examination of contractility in the combination of metformin and M_2_ and/or M_3_ receptor blocker treatment to investigate whether their action intersects revealed that the effect of muscarinic receptor blockers was potentiated by metformin, suggesting a synergistic action. This finding is important as parasympathetic tonus dominates throughout pregnancy until term;^35^ the attenuated CCh-induced contraction suggests the contribution of metformin to the silent myometrium state, which is required for implantation and safe development and maturation of the fetus until the time of delivery.

A major limitation of this study is not including myometrium samples from pregnant uterus and conducting experiments on normal uterine tissue. But being one of the first studies, it is believed that these findings will trigger and be the basis for further in vitro and in vivo studies.

Taken together, the results of the current study provide data about metformin-modulated myometrial contractility, giving clues about favorable reproductive benefits besides its indications and warranting further consideration of its use during pregnancy and in PCOS patients with fertility problems.

## Data Availability Statement:

The data that support the findings of this study are available on request from the corresponding author.

## Figures and Tables

**Table 1.** The Amplitude and Frequency of Spontaneous Contractions of Myometrium Strips and the Effect of Metformin Exposure

	Control	Metformin-Incubated	*P*
	**Spontaneous Contractions**		
	(n = 57)	(n = 57)	
Amplitude (% KCl-induced contraction)	38.85 ± 3.01	32.25 ± 3.01	.038
Frequency (Hz)	0.017 ± 0.002	0.006 ± 0.002	.023
	**Maximum Contraction to Oxytocin Stimulation**		
	(n = 8)	(n = 8)	
Amplitude (% KCl-induced contraction)	94.32 ± 5.21	41.32 ± 3.81*	.002
	**Maximum Contraction to Carbachol Stimulation**		
	(n = 8)	(n = 8)	
Amplitude (% KCl-induced contraction)	82.04 ± 3.54	64.83 ± 2.75*	.018

Data is presented as mean ± SEM.

*Statistically significant compared to control.

**Table 2.** The Maximum Force of Contraction (Emax) and Negative Logarithm of Molar Concentration That Results in 50% of Emax (pEC_50_) Values for Carbachol-Induced Contractions of Myometrium Strips in the Presence of Metformin and/or Muscarinic Receptor Antagonists: Methoctramine and 4-Diphenylacetoxy-N-Methylpiperidine

	Emax	pEC_50_
Control (n = 8)	76.57 ± 4.69	4.30 ± 0.09
Metformin (n = 8)	63.47 ± 3.92^*^	3.82 ± 0.05^*^
Methoctramine (n = 6)	67.32 ± 3.26^*^	3.64 ± 0.12^*^
4-DAMP (n = 7)	18.78 ± 2.08^**, ***, ****^	3.45 ± 0.08^*, ***, ****^
Metformin + Methoctramine (n = 6)	43.68 ± 3.14^**, ***, ****^	3.38 ± 0.11^*, ***^
Metformin + 4-DAMP (n = 7)	13.99 ± 1.09^**, ***, ****^	3.41 ± 0.08^*, ***, ****^
Metformin + Methoctramine + 4-DAMP (n = 7)	10.86 ± 0.91^**, ***, ****, #^	3.40 ± 0.07^*, ***, ****^

Data is presented as mean ± SEM.

4-DAMP, 4-diphenylacetoxy-N-methylpiperidine.

*P < .05 vs. control strips.

**P < .005 vs. control strips.

***P < .05 vs. metformin-incubated strips.

****P < .05 vs. methoctramine incubated strips.

^#^*P* < .05 vs. 4-DAMP incubated strips.
